# A Thoracic Outlet Syndrome That Concealed a Glioblastoma. Findings from a Case Report

**DOI:** 10.3390/medicina57090908

**Published:** 2021-08-30

**Authors:** Lorenzo Storari, Manuel Signorini, Valerio Barbari, Firas Mourad, Mattia Bisconti, Mattia Salomon, Giacomo Rossettini, Filippo Maselli

**Affiliations:** 1 Department of Neurosciences, Rehabilitation, Ophthalmology, Genetic and Maternal Infantile Sciences (DI-NOGMI), Campus of Savona—University of Genova, Via Magliotto 2, 17100 Savona, Italy; lorenzo.storari93@gmail.com (L.S.); valeriobarbari1993@gmail.com (V.B.); 2 Department of Radiology, ULSS 9 Scaligera, Mater Salutis Hospital, 37045 Legnago, Italy; manuel.signorini@gmail.com; 3 Department of Clinical Science and Translation Medicine, Faculty of Medicine and Surgery, University of Rome Tor Vergata, 00133 Rome, Italy; 50firas@gmail.com (F.M.); salomon.mattia@gmail.com (M.S.); 4 Department of Physiotherapy, LUNEX International University of Health, Exercise and Sports, L-4671 Differdange, Luxembourg; 5 Department of Medicine and Health Science “Vincenzo Tiberio”, University of Molise, c/o Cardarelli Hospital, C/da Tappino, 86100 Campobasso, Italy; mattia.bisconti@unimol.it; 6 School of Physiotherapy, University of Verona, 37100 Verona, Italy; giacomo.rossettini@gmail.com; 7 Sovrintendenza Sanitaria Regionale Puglia INAIL, 70126 Bari, Italy

**Keywords:** diagnosis, differential, glioblastoma, nervous system diseases, physical therapists, thoracic outlet syndrome

## Abstract

*Background:* Glioblastoma is the most frequent and aggressive malignant brain tumor among adults. Unfortunately, its symptoms can vary considerably depending on the size, location and the anatomic structures of the involved brain. *Case report:* A 58-year-old male amateur cyclist who suffered from sharp arm pain was examined for a thoracic outlet syndrome due to a previous clavicle fracture. Because of ambiguous results of the neck and nerve plexus imaging, he was referred to a neurosurgeon who properly suspected a brain tumor. The neuroimaging of the brain shown a 3 cm disploriferative mass with a blood enhancement within the left parietal lobe. The mass was urgently removed, and its histologic analysis stated a grade 4 glioblastoma. *Conclusion:* This case report highlights the differential diagnosis process and the teamwork approach needed to diagnose a rare presentation of a brain glioblastoma, which started its symptoms mimicking a thoracic outlet syndrome caused by a previous bone fracture.

## 1. Introduction

Shoulder pain is one of the most prevalent complaints seen both in outpatient clinics and emergency departments [1,2]. Unfortunately, the diagnostic process to frame the specific cause of shoulder pain is tricky, given that many adjacent anatomic structures could determine symptoms in the shoulder and whole upper limb regions [3,4]. In fact, in addition to musculoskeletal causes both nerves, blood vessels and visceral organs disorders can provoke symptoms in the shoulder, making the clinical reasoning process even more challenging [5,6]. Among them, the thoracic outlet syndrome (TOS) is noteworthy, which is a nonspecific diagnosis that refers to an array of conditions caused by compression of the neurovascular structures that pass through the thoracic outlet [7]. Particularly, there are three major types of thoracic outlet syndrome: (1) the compression of the artery by the first cervical rib or fibrous bands is known as arterial TOS, (2) the compression of the vein at the anterior costoclavicular junction is called venous TOS, and (3) the compression of the nerves (brachial plexus) at the scalene triangle, costoclavicular space and/or retropectoral space, is named neurogenic TOS [8]. Despite the fact that the incidence of TOS is questionable, a recent literature finding proves that neurogenic TOS seems to be the most frequent presentation, with an estimated incidence between 2 and 3 per 100,000 people per year [9]. This condition refers symptoms as numbness, paresthesias, strength weakness and fine motor dysfunction on the ipsilateral shoulder, arm and hand [10,11], which may be caused by traumas or repetitive load [12]. However, these symptoms may also be due to more severe conditions such as brachial plexus radiculopathies, cervical disorders or tumors [2]. In addition to these, brain and central nervous system (CNS) tumors such as meningiomas, pituitary tumors, and malignant gliomas are the most common adult brain tumor types [13]. Notably, glioblastomas and other malignant gliomas constitute nearly 75% of malignant brain tumors [14]. We reported the case of a man who complained of a tingling and sharp upper limb pain after a clavicular fracture, firstly investigated as neurogenic TOS, which turned out to be a grade IV glioblastoma.

## 2. Case Presentation

**Figure 1:**
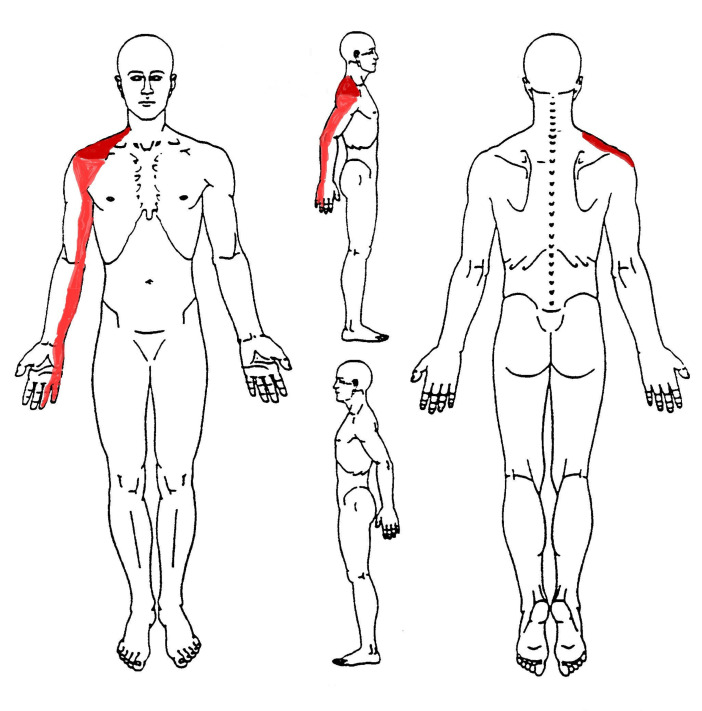
Body Chart. Bright red indicates the most painful body areas; pale red indicates the mild painful body areas.

A 58-year-old male amateur cyclist, manager in a company consulting firm, presented to outpatient physiotherapy clinic complaining of a tingling, numbness and burning pain wrapping all the right upper limb, from the hand to the supraclavicular region (see the body chart in Figure 1). Pain was stated with numeric pain rating (NPRS) scale [15], at 6/10 at the clavicle and shoulder, and 3/10 at the arm and at the IV/V° finger of the hand (Figure 1). The patient has 20 years of experience in cycling, and was usually training 2 days per week (120 km/week). The pain started 1 month ago from his right shoulder and clavicular fossa (NPRS 8/10), and then radiated down to the whole upper limb, while he started to train on roller due to the COVID-19 pandemic national lockdown. He experienced a few episodes of quick sharp pain at the clavicular fossa and the anterior deltoid region followed by numbness and weakness in the arm and forearm down to the hand. The latter began after 1 h of training on a roller, lasting about a couple of minutes and spontaneously resolved after some not-specific stretching movements of the head and shoulder, while still cycling but in a seated position without placing hands on the handlebar. Therefore, given that the symptoms were of short duration, without any limitation during training or working activity, the patient decided to carry out some self-managed stretching exercises to reduce the self-reported stiffness of the neck and shoulders. Nevertheless, the symptoms did not improve significantly, and for this reason, the patient self-presented to a physical therapist (PT).

### Investigations

During the history-taking, the patient reported to have had a fall during a bike tour one year before, from which he reported a midshaft displaced fracture of the right clavicle and 6 upper ribs (the exact localization of the latter was unavailable due to the loss of the chest plain radiograph by the patient) of the right side that were managed conservatively, as suggested by his orthopedic surgeon. Moreover, he denied any unexplained weight loss, history of malignancy, smoking and drinking habits, sleep disturbances or constitutional symptoms. After careful history-taking, a thorough MSK evaluation of neck, shoulder and scapular movements was performed. The diagnostic criteria of specific [16] and non-specific shoulder pain [4], cervical radiculopathy or radicular pain [17] were not completely fulfilled during the physical examination, for example: characteristics of the onset and type of pain; presence of any antalgic postures of neck and shoulder; painful movements of neck or shoulder; dermatomal distribution of the symptoms; clear neurological signs; positive upper limb nerve tension tests and compression tests. Therefore, the PT decided to perform a thorough examination of the thoracic outlet to evaluate a possible neurogenic TOS [18].

As stated by Povlsen et al. [19,20], the diagnosis process of TOS is currently treated as a diagnosis of exclusion, for this reason and to have outcome measures to be re-evaluated in the follow-up period, the QuickDASH [21] and the Cervical Brachial Symptom Questionnaire (CBSQ) [22] were administered to evaluate the features of the referred cervical brachial pain and shoulder pain. The questionnaires demonstrated a slight shoulder complaint during the prolonged working and sports activities, described with tingling and numbness sensation (83/95 on QuickDASH), and a pattern of mild and random sensory disturbance and motor weakness localized to the lower trunk of the brachial plexus (100/120 on CBSQ). Given that the pathway of symptoms was faint and not constant, mostly related to maintained postures such as holding the handlebar and working on the laptop, the PT decided to further evaluate the possible TOS using the frame proposed by Illig et al. [9]. This physical examination frame highlighted painful tender point at the base of the neck (upper trapezius and anterior scalene), the shoulder/upper arm (anterior deltoid) and chest (minor pectoralis), and numbness/paresthesia at the upper arm, forearm and hand. Moreover, the Elevated Arm stress Test [9] (which is a sort of modified Roos stress test) resulted positive and a grip test for the IV and V fingers were slightly weak compared to the left side. In line with the 2016 guideline reporting standards of the Society for Vascular Surgery TOS, the PT related the patient symptoms to a neurogenic TOS; furthermore, to exclude the possible vascular conditions of TOS, he performed the Halstead maneuver, Wright’s test, and Cyriax Release test [23], which both resulted slightly positive. Taking account all the signs and symptoms, the PT suspected a neurogenic TOS and referred the patient to his General Practitioner (GP) for a medical examination and pathway management process. The GP confirmed the diagnostic hypothesis of neurogenic TOS and prescribed physical therapy and a Magnetic Resonance Imaging (MRI) of the cervical spine and brachial plexus with and without contrast dye. Concurrently, the patient started a program with the PT, based on manual therapy for the tender point of the neck and shoulder, and exercise therapy to strengthen the postural muscles of the trunk, neck, and shoulder to improve the comfort during cycling and working activity.

## 3. Results

**Figure 2:**
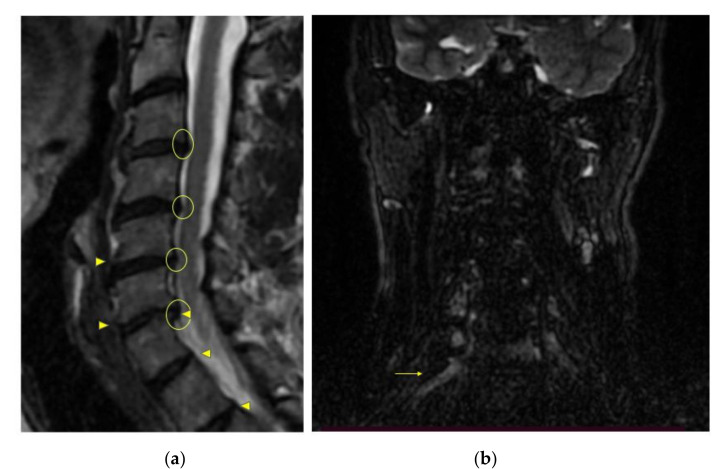
Cervical spine and Brachial Plexus MRI. (**a**) Sagittal T2 weighted image of the cervical spine. Straightening of the lordosis with spondylodiscoartrosic changes in C3-T1 tract are shown. The most evident features are presence of enthesophytes (arrowheads) both anteriorly and posteriorly, where they cause a reduction in intervertebral space height and moderate bulging anulus imprinting CSF column (yellow circles). (**b**) Coronal STIR image of the cervical spine. Right-sided hyperintensity aspect of the inferior trunk and medial cord of brachial plexus (yellow arrow) at C8-T1 level, indicating edema at this level, corresponding to the ulnar nerve symptoms.

The MRI reported a straightening of the cervical lordosis, widespread spondyloarthrosis of the cervical zygapophyseal joint, wide bulging disks at C5-C6 and C6-C7 level and signal hyperintensity on the right primary brachial plexus trunks, for the detailed imaging, see Figure 2. Moreover, the radiologist suggested performing an electromyography (EMG) for deepening the diagnostic process. It is noteworthy that the EMG of the brachial plexus did not report any alteration of the nerve conduction. The PT performed three manual therapy sessions of soft-tissue mobilization techniques (three sessions every other day in a week) to reduce the tenderness in the neck, shoulder and chest painful muscles [24,25] and six exercise sessions (two sessions per week for three weeks) as proposed by Levine et al., for the conservative treatment of the neurogenic TOS [26].

### 3.1. Differential Diagnosis

However, the episodes of quick and burning pain became more frequent, even during light normal activities, such as washing the dishes, and further worsened (NPRS 8/10 at the clavicle and shoulder, and 5/10 at the arm and at the IV/V° finger of the right hand) during the physical therapy program. For this reason, after the MRI and EMG examination, the PT, in accordance with the GP, referred the patient to his orthopedic surgeon for a detailed evaluation of the clavicle movements as a possible cause of the neural symptoms. The orthopedic surgeon stated that the clavicle was likely not the cause of the worsening of the symptoms, while the latter was probably related to the cervical spine bulging disks of C5-C6 and C6-C7 levels. Moreover, the day after the orthopedic examination the patient experienced a tonic-clonic seizure over his forearm and IV/V° finger muscles, under PT’s supervision. The seizure started while the patient was performing a Serratus Punch exercise [27] for scapular protraction and serratus anterior strengthening. The seizure lasted about 1 min and was self-resolved by keeping the upper limb down by the side. The PT recognized the signs of a possible nervous system red flag as described in the OSPRO-ROS tool [28,29], and after warning the GP and the orthopedic surgeon by telephone, he referred the patient to a neurosurgeon.

**Figure 3:**
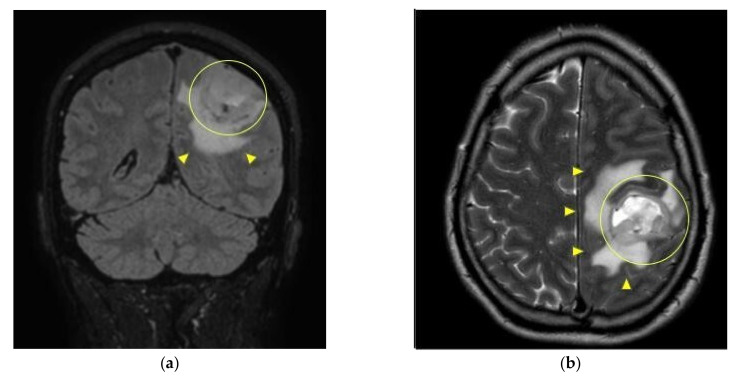

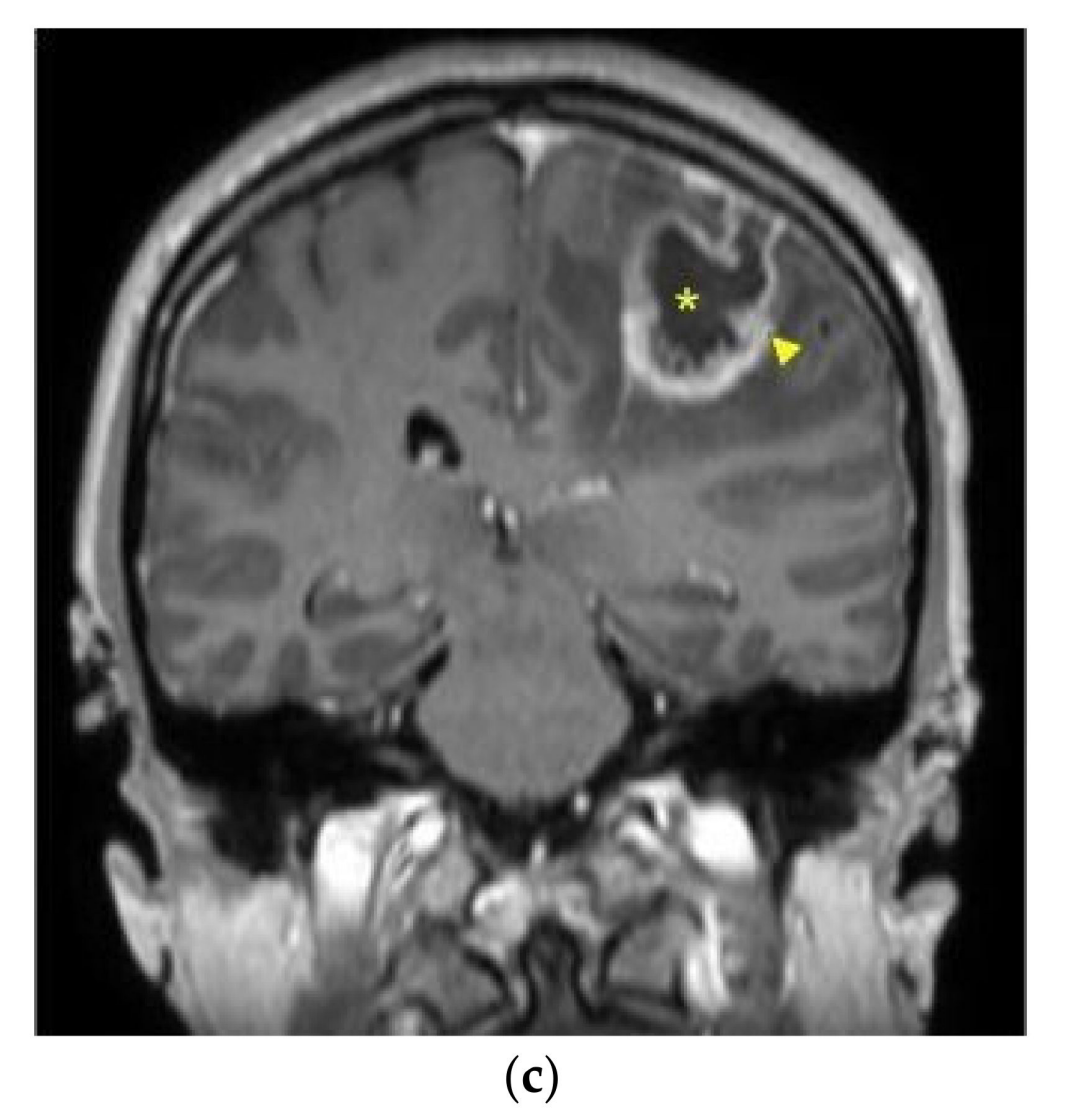
Brain MRI. A 3 cm left parietal solid mass (circle) surrounded by edema is demonstrated at brain MRI; its characteristics are typical for gliobastoma. Coronal FLAIR image (T2-weighted with CSF suppression; (**a**) and T2-weighted axial image (**b**) show hyperintense lesion (circle) with heterogeneous signal because of hemorragic intralesional foci and flow voids, surrounded by edema (arrowhead). In (**b**) both solid peripheral ring and necrotizing/hemorragic intralesional foci are better depicted. Coronal T1-weighted image after contrast (**c**) shows peripheral and irregular enhancement with nodular components (arrowhead), that surround necrosis (asterisk).

Two days after, during the neurosurgeon assessment, the patient also reported experiencing a 30 s epileptic tonic-clonic seizure episode, which occurred the evening before, while he was watching TV, which was recorded using the phone by the patient itself (for a detailed description, see the video in Supplementary Materials). He described in detail the episode and other symptoms to the neurosurgeon, who decided to prescribe an urgent brain MRI with and without contrast enhancement, and an electroencephalography (EEG) to evaluate a possible epileptic seizure [30]. Unfortunately, the MRI showed a 3 cm mass within the left parietal lobe with blood enhancement, that probably has a compressive action on the brain ventricle and features of glial cell disproliferative lesion (see Figure 3).

### 3.2. Treatment

**Figure 4:**
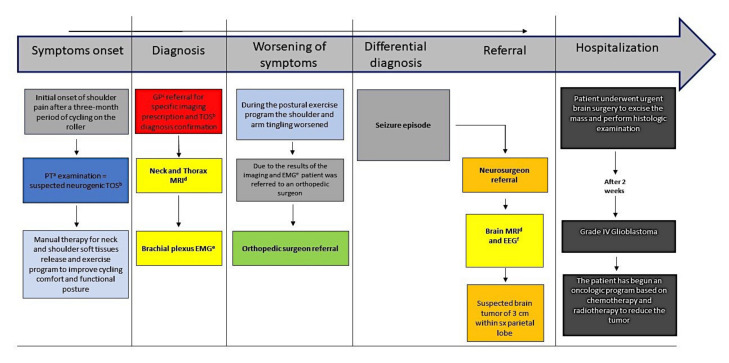
Timeline.

After a detailed evaluation of the brain MRI, the neurosurgeon obtained the patient-informed consent to a surgical craniotomy for maximal safe resection of excide the brain mass, in order to perform the histological diagnosis. The mass was mostly removed, and the resection of the abnormal tissues provoked an ataxic gait, a mild postural imbalance (positive Romberg’s test [31]) and loss of sensitiveness in lateral area of the right leg and foot, and a loss of motor coordination in the ulnar nerve muscles of the right hand. The result of histological examination was a grade IV glioblastoma multiforme. After the surgery, the patient started a chemotherapy (temozolomide 135 mg, every day, once a day for six weeks) and radiotherapy program (five sessions a week, for six weeks) to reduce the remaining portions of the tumoral mass, and a physical therapy program to improve the functional neurological deficits. For a detailed description of the timeline management, see Figure 4.

### 3.3. Outcome and Follow-Up

At the second postoperative month, the patient has completed the first cycle of chemotherapy and radiotherapy without major side-effects and restored the physiological gait pattern and balance control, while the loss of sensitivity in the right lower limb and hand was still present.

## 4. Discussion

To the best of the authors’ knowledge, this is the first case report that highlights the differential diagnosis process of a brain glioblastoma in which symptoms were mimicking the symptoms of a neurogenic TOS. As clearly reported in the scientific literature, the diagnosis of TOS is challenging [19,20], because a definitive consensus in diagnostic criteria has not yet been established [32], and the several possible causes of TOS could be related both to vascular and neurological conditions that provoke compressive stress on the brachial plexus [10,11]. Among these, as in the present case, falls and traumas such as cervical whiplash and fractures of the clavicle [33], or maintained stressful positions of the shoulder range of motion [34] have been reported to be risk factors of TOS. Unfortunately, these events are very common [35,36], and there is no direct causal association with TOS [37], so this could explain the poor awareness among doctors and healthcare professionals toward TOS and a substantial lack of consensus in diagnosis that makes it difficult to assess between physiologic brachial plexus compression and the true syndrome [18], as endorsed by the vague epidemiological available data on this condition [9]. Furthermore, the poor diagnostic accuracy of clinical tests for TOS [38], and its broad localization and features of symptoms, both vascular and neurogenic are very similar to other musculoskeletal, neurological and visceral disorders [12,39]. In some cases, MRI with dynamic maneuvers can detect anatomical abnormalities potentially responsible for compression; indeed, specificity is sufficiently high to provide guidance especially for planning the surgical procedures; however, sensitivity is too low for MRI to be useful as a single screening test [40].

In this regard, to improve the clinical reasoning in the case of differential diagnosis, more attention should be paid to the differentiation between peripheral and central neurological symptoms. In the present case, notwithstanding the careful patient history-taking, and even though both the PT and GP performed a thorough examination of the neck and upper limb movements [16] and a comprehensive neurological evaluation of the cervical roots and brachial plexus tension tests [17,23], there were no clear signs that suggested such a central neurological disorder. When the episode of seizure occurred, rather, it was possible to understand that weakness of the IV-V° finger of the right hand was related to the bulging disks in the cervical spine and the plexopathy highlighted by the MRI, whereas the quick episodes of burning pain, numbness and tingling in the upper limb were the initial focal seizures [41]. As highlighted in a recent clinical practice guideline, these signs are possible symptomatic epileptic episodes secondary to gliomas in adult population [42]. Epileptic seizures often present as the first clinical symptom of brain tumors and probably the most common presentation of such diseases [43,44]. To properly frame the diagnostic process of seizures, in order to distinguish between idiopathic or glioma-related epilepsy, it is necessary to perform a comprehensive history-taking, detailed physical examination and the most suitable imaging [45] within the assessment of patients complaining of TOS-like symptoms. Unfortunately, the etiology of seizures, similar to most of the red flags [46], is tough and often manifold to identify among acute or remote causes [47], but brain tumors as glioblastomas, which are the most common malignant type of CNS tumor [14], commonly determine such episodes [48].

Glioblastoma is a deeply aggressive and infiltrative malignant tumor that has the characteristics of a unique site of origin, the brain [49]. Furthermore, an important hallmark of glioblastomas is their heterogeneity, which is intended both at cellular and molecular level and makes them resistant to the most of the chemotherapy strategies available [50,51]. In fact, it is well known that the blood–brain barrier is a hurdle for the immunotherapy of any pathological disorder in the brain [51,52].

It is noteworthy, glioblastoma shows wide variability in its presentation, location, and patient demographics, overlapping in many ways with a wide variety of neoplastic or non-neoplastic lesions [53]. This overlap unfortunately can extend to additionally include molecular alterations and even clinical and radiologic features [53]. Some of these entities that represent primary CNS neoplasms (such as lymphoma, oligodendroglioma or anaplastic astrocytoma) are usually of a lower WHO grade and may even be non-neoplastic lesions (such as cerebral access or stroke, toxoplasmosi, encephalitis and seizure disorder), and have a wide range of prognostic and treatment implications [49].

In contrast, these other entities should not be mistaken for a glioblastoma, treatment of which in most patients, as well as in the present case, starts with neurosurgery, either resection or biopsy, which are fundamental both for histological diagnosis and to reduce the tumor mass [53]. Due to the high incidence of glioblastomas in adult population [13,14] both physicians and health professionals, particularly physical therapists, should evaluate every alarming symptom that it is supposed to be reliable for a CNS disorder.

For this reason, it is essential for healthcare professionals to be aware of the clinical meaning of particular and atypical clinical manifestations [54,55,56,57,58], and to understand in detail the seizure episodes and their presentation pattern, as in our case report. Furthermore, early detection and proper management of seizures can reduce complications and costs caused by unnecessary treatment [42]. Consequently, this case reports highlights the importance of improving the diagnostic process of pathologies such as neurogenic TOS, which may occur with signs and symptoms similar to more serious conditions such as glioblastomas and other CNS tumors, in order to focus attention on the need for clearer diagnostic criteria and to properly refer the patient to the more appropriate pathway of care. This case report also underpins the value of multidisciplinary healthcare teamwork approach during the differential diagnosis process of a rare presentation of a common disease.

Unfortunately, the substantial absence of clear reference standards related to the diagnosis of TOS make this case report an attention-grabbing paper toward the need of a thorough differential diagnosis process in those patients with suspicious and doubtful symptoms, rather than a detailed description of how to manage properly patients with TOS symptoms.

## 5. Conclusions

Differential diagnosis is a key element of any healthcare professionals’ clinical reasoning, especially if the supposed disease is challenging to diagnose, as with TOS. In fact, framing each sign and symptom in the case of TOS is difficult given that the diagnosis often requires several professionals. Therefore, applying a teamwork diagnostic process, PTs, GPs and medical specialists are able to carry out a comprehensive clinical evaluation of different MSK affections and to screen doubtful conditions that could require an appropriate pathway of care.

## Data Availability

Not applicable.

## Supplementary Materials

The following are available online at https://www.mdpi.com/article/10.3390/medicina57090908/s1, Video S1: Epilectic tonic-clonic seizure.
